# Dynamic relationships among first-year university students’ critical thinking, academic self-concept, and student engagement: a cross-lagged study

**DOI:** 10.3389/fpsyg.2026.1846917

**Published:** 2026-06-17

**Authors:** Yuzhen Gao, Runjie Jiang, Keshun Zhang, Shuang Song

**Affiliations:** 1 School of Marxism, Qingdao University, Qingdao, China; 2 School of Education, Nanjing University, Nanjing, China; 3 Department of Psychology, Qingdao University, Qingdao, China; 4 School of Foundational Education, University of Health and Rehabilitation Sciences, Qingdao, China

**Keywords:** academic self-concept, critical thinking, first-year university student, longitudinal study, student engagement

## Abstract

Critical thinking and academic self-concept are key predictors of student engagement among first-year university students. Yet, the mechanisms underlying their relationships remain unclear. This one-year longitudinal study examined cross-lagged relationships between critical thinking and academic self-concept and their effects on student engagement. A sample of 3,437 Chinese first-year university students completed measures at two waves; data were analyzed using structural equation modeling (SEM). Four main findings emerged. First, critical thinking and academic self-concept showed significant cross-lagged effects: critical thinking negatively predicted academic self-concept, whereas academic self-concept positively predicted critical thinking. Second, critical thinking positively predicted T2 student engagement. Third, academic self-concept negatively predicted T2 student engagement. Fourth, these dynamic relationships were consistent across Math and English disciplines. The findings extend the literature on student engagement and offer insights into its development during the transition to university.

## 1 Introduction

Student engagement is critical to the academic adjustment of first-year university students. The transition to university typically involves substantial changes in learning and living environments; thus, stronger engagement can help students affirm their sense of value and adapt to new demands (Martinie and Shankland, 2024). Early engagement supports academic adjustment, competence development, and longer-term academic growth (Fredricks et al., 2004; Kahu, 2013; Wang and Eccles, 2013). It is also a key indicator of learning status and quality (Zhen et al., 2017). Therefore, understanding how to promote engagement among first-year students is therefore essential for improving adjustment and outcomes.

Social cognitive theory provides a foundation for understanding these processes: cognition shapes behavior (Bandura, 1991). As a manifestation of learning behavior, engagement is influenced by cognitive factors. When individuals mobilize cognitive resources for self-evaluation and develop a positive self-concept, they experience favorable cognitive states that enhance engagement (Schnitzler et al., 2021). Building on this framework, the present study focuses on how critical thinking (a higher-order cognitive skill) and academic self-concept (a self-evaluative academic belief) jointly shape student engagement during the early stages of university.

Extant research links higher-order thinking with engagement (Greene, 2015), with critical thinking playing a central role (Appleton et al., 2008). Critical thinking involves interpreting, analyzing, evaluating, and refining one’s reasoning regarding evidence, concepts, and methods (Pascarella and Terenzini, 2005). These abilities support the development of both self-concept and engagement (Phan, 2009; Mafarja and Zulnaidi, 2022). In parallel, academic self-concept, students’ evaluation of their academic abilities, can bolster confidence and strengthen motivation to engage (Dwyer et al., 2017), facilitating cognitive development and higher levels of engagement (Galugu and Samsinar, 2019; Wang and Wu, 2008). Accordingly, the present study examines the reciprocal relations among critical thinking, academic self-concept, and student engagement, while taking potential disciplinary differences between Math and English into account.

### 1.1 Student engagement

Student engagement originated from Astin’s (1984) theory of student involvement and was later conceptualized as a multidimensional construct (Kuh, 2001). Fredricks et al. (2004) integrated these perspectives and defined engagement as a meta-construct comprising behavioral, emotional, and cognitive dimensions. Behavioral engagement refers to constructive classroom participation and the avoidance of disruptive behaviors. Emotional engagement reflects positive affect (e.g., interest, enjoyment) and the regulation of negative emotions (e.g., anxiety, frustration; Zhang et al., 2021). Cognitive engagement reflects the investment of mental effort in learning (Chiu, 2021). Engagement predicts motivation and learning quality; students with higher engagement are more likely to adopt adaptive strategies and manage academic stress, thereby improving skills and performance (Wang and Wang, 2025). Conversely, low engagement is associated with fewer resources and less effective coping, leaving students more vulnerable to negative academic emotions and adjustment difficulties (Liu et al., 2024). Meta-analytic evidence links engagement with achievement, learning experience, and satisfaction (Wang and Wang, 2021), and with self-directed learning and interest (Chukwuedo et al., 2021). Importantly, student engagement is not only multidimensional but also partly subject-specific. Prior research suggests that students may engage differently across subject domains because learning tasks, classroom interactions, and performance demands vary by discipline (Weich et al., 2024). For example, engagement in Math is often reflected in cognitive effort, persistence, and task-focused participation in problem solving, whereas engagement in English or language-related subjects is more often manifested through reading, discussion, verbal participation, and interaction (Ingram, 2013; Papageorgiou et al., 2025; Svalberg, 2009). Thus, understanding engagement among first-year students is crucial for effective adjustment and holistic development.

### 1.2 Critical thinking and student engagement

Critical thinking is a higher-order cognitive ability involving the interpretation and evaluation of evidence, concepts, and methods, and the refinement of reasoning (Pascarella and Terenzini, 2005). As a key competency for university students, it facilitates short-term decision making and long-term intellectual development (Astleitner, 2002; Halpern, 2014; Terenzini et al., 1995). From a social-cognitive perspective, higher-order strategies increase the need for cognition and deepen immersion in learning activities, a key pathway to engagement and academic success (Whitney et al., 2016). Critical thinking enhances deep processing, reflective judgment, and problem solving, which in turn bolster cognitive, emotional, and behavioral engagement (Huang et al., 2025; Manousou, 2025; Phan, 2009). Empirically, Álvarez-Huerta et al. (2023) report a strong association between critical thinking and engagement, and longitudinal evidence shows that earlier critical thinking predicts later engagement (Lv et al., 2022). Consistent with these mechanisms, curricula explicitly designed to cultivate critical thinking have been shown to increase student engagement (Caratozzolo et al., 2019). Longitudinal evidence indicates that prior critical thinking predicts subsequent engagement (Lv et al., 2022). While the theoretical link between critical thinking and engagement is well established, this dynamic interplay may not operate uniformly across educational contexts. In particular, the expression and application of critical thinking are often domain-specific. In Math, critical thinking is often embedded in logical reasoning and structured problem solving, whereas in English or language learning, it is more explicitly reflected in interpretation, argumentation, and critique (Sachdeva and Eggen, 2021; Sukmojati et al., 2025). These disciplinary differences suggest that the way critical thinking translates into student engagement may vary across academic subjects.

*Hypothesis 1 (H1)*: Critical thinking positively predicts T2 student engagement.

### 1.3 Academic self-concept and student engagement

Academic self-concept refers to students’ self-perceptions in academic contexts and includes cognitive, affective, and evaluative components (Goetz et al., 2012). It predicts interest, choices, performance, and achievement (Durik et al., 2006). The participation-identification model posits that engagement is shaped by cognitive appraisals (Finn, 1989), and research generally supports a positive association between self-concept and engagement (Guo et al., 2017; Galugu and Samsinar, 2019). Academic self-concept elevates expectations of success and perceived task value, thereby strengthening intrinsic motivation and participation (Eccles and Wigfield, 2020), reducing anxiety, and increasing emotional engagement (Pekrun, 2017). In contrast, low self-concept is associated with burnout (Bassi et al., 2007), and experimental studies show causal links between self-concept and engagement (Llorens et al., 2007). Consequently, a positive academic self-concept not only fosters engagement via enhanced self-efficacy (Linnenbrink and Pintrich, 2003), but also stimulates intrinsic motivation and promotes more positive learning-related emotions (Pekrun, 2006). In turn, these processes spontaneously increase engagement across behavioral, cognitive, and emotional dimensions (Pekrun and Linnenbrink-Garcia, 2012). Furthermore, academic self-concept is also widely regarded as domain-specific rather than purely general. Students may perceive themselves as more competent in one subject than in another, partly because self-evaluations are shaped by dimensional comparison processes across domains (Marsh and Martin, 2011; Möller and Marsh, 2013). Consistent with this view, research has shown that Math self-concept and reading self-concept primarily predict outcomes within their corresponding domains (Susperreguy et al., 2018). This subject specificity also implies interests to test whether the association between academic self-concept and engagement is identical or not across Math and English.

*Hypothesis 2 (H2)*: Academic self-concept positively predicts T2 student engagement.

### 1.4 Critical thinking and academic self-concept

Social cognitive theory posits that the use of cognitive strategies is shaped by individuals’ cognitive levels, while these strategies in turn influence cognitive outcomes (Bandura, 1989). Critical thinking and academic self-concept may form dynamic reciprocal relations.

Critical thinking can strengthen academic self-concept because it helps students evaluate learning content, monitor their understanding, and make more accurate judgments about their own academic competence (Stupnisky et al., 2008; Phan, 2010). Through analysis and reflection, students are more likely to base self-evaluations on actual performance rather than external feedback, which in turn promotes a clearer and more stable academic self-concept. Empirical studies have also reported a positive association between critical thinking and academic self-concept (Truxillo et al., 2008; Cormier et al., 2010; Mafarja and Zulnaidi, 2022).

Conversely, academic self-concept may foster critical thinking. Students who perceive themselves as academically capable are more confident in dealing with challenging tasks and are therefore more willing to engage in higher-order cognitive activities such as analysis, reasoning, and evaluation (Eccles and Wigfield, 2020; Dwyer et al., 2017). In contrast, students with lower academic self-concept may avoid demanding tasks, which constrains the development of critical thinking. Prior research further suggests that academic self-concept predicts deeper cognitive engagement and subsequent critical thinking (Marsh and Martin, 2011; Martin and Liem, 2010; Phan, 2014). Taken together, these findings suggest a dynamic reciprocal relationship between critical thinking and academic self-concept. Because both critical thinking and academic self-concept are likely to be shaped by subject-specific learning demands and evaluative standards, this reciprocal association may unfold differently across disciplinary contexts such as Math and English.

*Hypothesis 3 (H3)*: Critical thinking and academic self-concept reciprocally predict each other over time.

Although prior research has shed light on the relationships among critical thinking, academic self-concept, and student engagement (e.g., Lv et al., 2022; Llorens et al., 2007; Galugu and Samsinar, 2019), three limitations remain. First, student engagement has been studied mainly in relation to environmental and instructional factors, while the roles of cognitive skills and self-concept have received less attention, despite growing evidence of their importance (Schnitzler et al., 2021). Second, critical thinking and academic self-concept have typically been treated as separate factors, rather than examined within an integrated framework, even though they are theoretically interconnected (Cormier et al., 2010; Wang and Wu, 2008). Third, existing research has been conducted largely within specific disciplinary contexts, making it difficult to determine whether the relationships among critical thinking, academic self-concept, and student engagement are generalizable across disciplines (Weich et al., 2024; Marsh and Yeung, 1998; Möller et al., 2011).

To address these gaps, the present study examined (i) the cross-lagged associations between critical thinking and academic self-concept among first-year university students, while also (ii) investigating whether critical thinking and academic self-concept predicted T2 student engagement over time. Specifically, this study explored (iii) whether the longitudinal relationships among critical thinking, academic self-concept, and student engagement are consistent across different subject domains (specifically, Math and English).

By integrating cognitive skills and self-belief systems within a longitudinal framework, this study clarifies how critical thinking and academic self-concept jointly and dynamically drive student engagement over time. The anticipated findings also carry practical implications for higher education: rather than focusing solely on environmental factors, a deeper understanding of these internal mechanisms may inform curriculum and instructional design that simultaneously cultivates critical thinking and strengthens academic self-concept. Such an approach offers a sustainable strategy to enhance engagement and mitigate academic burnout across subject domains.

## 2 Methods

### 2.1 Ethical statement

This study complied with the ethical standards of the Declaration of Helsinki. All procedures were approved by our university’s Research Ethics Committee. Before data collection, participants were informed of the study’s purpose, the voluntary nature of participation, confidentiality, and their right to withdraw at any time without penalty. Participants aged 18 years or older provided their own informed consent. For those under 18 years, informed consent was obtained from a legal guardian and assent from the student. No personally identifiable information was collected or reported.

### 2.2 Participants and design

Data were collected from a comprehensive university in China across two waves within one academic year (T1: September; T2: May of the following year). We used convenience sampling and recruited from a student body representing 31 provinces, providing broad geographic diversity. Critical thinking and academic self-concept were assessed at both waves, whereas student engagement was assessed at T2 only. A total of 3,437 students participated in Time 1 (T1); 1,777 completed both two waves (48.30% attrition; 51.70% completion). Participants’ ages ranged from 15 to 28 years (*M* = 18.31, *SD* = 0.76), and 45.77% were female (*N* = 1,573). Attrition analyses compared completers with noncompleters (participants missing T2). To assess potential attrition bias, we compared participants who completed all waves with those who dropped out. Independent-samples t tests and chi-square tests showed no significant baseline differences in gender, critical thinking, academic self-concept, or student engagement (all *p*s > 0.05). Thus, there was no evidence that structural relations among study variables differed between groups. Full information maximum likelihood (FIML) was used to handle missing data (Enders, 2022).

### 2.3 Measures

#### 2.3.1 Student engagement

Student engagement was assessed using the ten-item student engagement scale (Skinner et al., 2009), with five behavioral items (e.g., “I do my best to learn in class”) and five affective items (e.g., “I fully engage when we do something in class”). Items were rated from 1 (strongly disagree) to 4 (strongly agree). Confirmatory factor analyses (CFAs) indicated good fit for both English (*χ*^2^(22) = 224.74, CFI = 0.99, TLI = 0.99, RMSEA = 0.05, SRMR = 0.01) and Math (*χ*^2^(15) = 143.31, CFI = 0.99, TLI = 0.99, RMSEA = 0.05, SRMR = 0.01). Cronbach’s alpha was 0.96 for Math and 0.97 for English.

#### 2.3.2 Critical thinking

Students’ critical thinking was assessed using the critical thinking scale (Stupnisky et al., 2008). This scale has five items (e.g., “When a theory, interpretation, or conclusion is presented in class or in readings, I try to judge whether there is good evidence supporting it”). Items were rated on a 7-point Likert scale ranging from 1 (strongly disagree) to 7 (strongly agree), with higher scores indicating higher levels of critical thinking. CFAs indicated good fit for both English (*χ*^2^(2) = 4.47, CFI = 1.00, TLI = 0.99, RMSEA = 0.02, SRMR = 0.004) and Math (*χ*^2^(3) = 6.500, CFI = 1.00, TLI = 0.99, RMSEA = 0.02, SRMR = 0.004). Cronbach’s alphas were 0.84 and 0.95 for Math at the two time points and 0.86 and 0.96 for English at the two time points.

#### 2.3.3 Academic self-concept

Academic self-concept was assessed using the four-item academic self-concept scale (Goetz et al., 2010), including three positively worded items (e.g., “I got good grades in Math/English class”) and one negatively worded item (e.g., “I am hopeless in Math/English class”). Items were rated on a 5-point Likert scale ranging from 1 (strongly disagree) to 5 (strongly agree). After removing one item with a factor loading below 0.40 (Stevens, 2002), CFAs indicated good fit (English: *χ*^2^(0) = 0.000, CFI = 1.000, TLI = 1.000, RMSEA = 0.000, SRMR = 0.000; Math: *χ*^2^(0) = 0.000, CFI = 1.000, TLI = 1.000, RMSEA = 0.000, SRMR = 0.000). Cronbach’s alphas were 0.88 and 0.94 for Math at the two time points and 0.93 and 0.95 for English at the two time points.

### 2.4 Analytic strategy

Analyses proceeded in three steps. First, descriptive statistics, correlations, and t tests were conducted in SPSS 26.0. Second, cross-lagged panel structural equation models were estimated in Mplus 8.3 to examine longitudinal relationships between critical thinking and academic self-concept, as well as the predictive associations of T1 critical thinking and T1 academic self-concept with T2 student engagement. Third, these associations were compared across Math and English using multiple-group SEMs. In this study, robust maximum likelihood estimation (MLR) was used for model estimation, and full information maximum likelihood (FIML) was used to handle missing data (Enders, 2022). Missing data were estimated using the expectation–maximization (EM) algorithm in SPSS. This method uses an iterative maximum-likelihood procedure to estimate missing values based on the observed data.

## 3 Results

### 3.1 Common method bias

A Harman single-factor test (Podsakoff et al., 2003) was conducted across all items. Seven factors with eigenvalues greater than 1 were extracted; the largest accounted for 39% of the variance, below the 40% threshold, suggesting common method variance was not a major concern.

### 3.2 Descriptive statistics and correlations

Descriptive statistics and bivariate correlations are reported in Table 1. In Math, critical thinking, academic self-concept, and student engagement at T1 and T2 were positively correlated with one another (*rs* = 0.33 to 0.72, *p*s < 0.001). In English, these variables were also positively intercorrelated (*r* = 0.19 to 0.71, *p*s < 0.001). The *t*-test results indicated that first-year students’ academic self-concept increased significantly after college entry in both Math, |*t*| (3436) = 10.24, *p* < 0.001, Cohen’s *d* = 0.17, and English, |*t*| (3436) = 17.66, *p* < 0.001, Cohen’s *d* = 0.24. Critical thinking also increased significantly over 1 year in Math, |*t*| (3436) = 117.63, *p* < 0.001, Cohen’s *d* = 2.02, and English, |*t*| (3436) = 104.09, *p* < 0.001, Cohen’s *d* = 1.81.

**Table 1:** Descriptive statistics and correlations among variables (*N* = 3,437).

Variable	1	2	3	4	5	6	7	8	9	10	11	12
1. T1 Math critical thinking	—											
2. T1 Math academic self-concept	0.60^***^	—										
3. T2 Math critical thinking	0.50^***^	0.38^***^	—									
4. T2 Math academic self-concept	0.45^***^	0.72^***^	0.54^***^	—								
5. T2 Math student engagement	0.36^***^	0.33^***^	0.62^***^	0.50^***^	—							
6. T1 English critical thinking	0.36^***^	0.07^***^	0.29^***^	0.05^**^	0.27^***^	—						
7. T1 English academic self-concept	0.12^***^	−0.01	0.13^***^	−0.06^**^	0.19^***^	0.66^***^	—					
8. T2 English critical thinking	0.32^***^	0.18^***^	0.68^***^	0.29^***^	0.52^***^	0.49^***^	0.41^***^	—				
9. T2 English academic self-concept	0.05^**^	−0.06^**^	0.27^***^	0.04^*^	0.32^***^	0.45^***^	0.71^***^	0.57^***^	—			
10. T2 English student engagement	0.21^***^	0.11^***^	0.48^***^	0.24^***^	0.70^***^	0.43^***^	0.41^***^	0.67^***^	0.58^***^	—		
11. Discipline	−0.09^***^	−0.05^**^	−0.22^***^	−0.16^***^	−0.35^***^	−0.08^***^	−0.07^***^	−0.26^***^	−0.19^***^	−0.44^***^	—	
12. Gender	−0.17^***^	−0.11^***^	−0.04^*^	−0.05^**^	0.15^***^	0.11^***^	0.17^***^	0.09^***^	0.13^***^	0.21^***^	0.09	—
*M*	3.34	3.05	4.95	3.16	3.16	3.38	3.11	4.89	3.32	3.20	—	—
*SD*	0.74	0.93	0.85	0.83	0.44	0.77	0.99	0.89	0.78	0.43	—	—

^*^p < 0.05, ^**^p < 0.01, ^***^p < 0.001. Gender was coded as 0 = female and 1 = male. Discipline was coded as 0 = English and 1 = Math.

### 3.3 Model comparison

To examine longitudinal relationships among critical thinking, academic self-concept, and student engagement, a series of competing SEMs were tested (Table 2). Model 1 included autoregressive paths for critical thinking and academic self-concept, their within-time correlations, and T2 predictions of student engagement. Model 2 added the path from critical thinking to academic self-concept. Model 3 added the path from academic self-concept to critical thinking. Model 4 included both cross-lagged paths. Model 5 further added predictive paths from T1 critical thinking and academic self-concept to T2 engagement. Model comparisons indicated that Model 5 provided a significantly improved fit over the preceding nested model and was therefore retained as the final model. Compared with Model 4, Model 5 showed significant improvement in model fit for both Math, Δ*χ*^2^(2) = 12.00, *p* < 0.01, and English, Δ*χ*^2^(2) = 35.17, *p* < 0.01, while maintaining good overall fit indices.

**Table 2:** Model fit and comparison results (*N* = 3,437).

Model	*df*	*χ* ^2^	CFI	TLI	RMSEA	Comparisons	Δ*χ*^2^	Δdf	*p*
Math									
Model 1	288	1246.39	0.98	0.97	0.03	M2-M1	2.22	1	>0.05
Model 2	287	1244.17	0.98	0.97	0.03	M3-M1	6.87	1	<0.01
Model 3	287	1239.52	0.98	0.97	0.03	M4-M1	11.60	2	<0.01
Model 4	286	1234.79	0.98	0.97	0.03	M4-M2	9.38	1	<0.01
Model 5	284	1222.79	0.98	0.97	0.03	M4-M3	4.73	1	<0.05
						M5-M4	12.00	2	<0.01
English									
Model 1	290	1352.35	0.98	0.97	0.03	M2-M1	12.44	1	<0.01
Model 2	289	1339.91	0.98	0.97	0.03	M3-M1	4.94	1	<0.05
Model 3	289	1347.41	0.98	0.97	0.03	M4-M1	24.12	2	<0.01
Model 4	288	1328.23	0.98	0.97	0.03	M4-M2	11.68	1	<0.01
Model 5	286	1293.06	0.98	0.97	0.03	M4-M3	19.18	1	<0.01
						M5-M4	35.17	2	<0.01

^*^p < 0.05, ^**^p < 0.01, ^***^p < 0.001.

### 3.4 Cross-lagged relationships among critical thinking, academic self-concept, and student engagement

Cross-lagged SEMs were estimated separately for Math and English. Given observed associations with gender and subject, these variables were included as controls. A collinearity diagnostic analysis was conducted for the predictor variables in the Math and English models. The results showed that the VIF values ranged from 1.643 to 2.657 in the Math model and from 1.700 to 2.931 in the English model, all of which were below the commonly used threshold of 5. These results indicate that there were no serious multicollinearity issues in either model, supporting the reliability of the SEM path estimates.

#### 3.4.1 Math model

**Figure 1:**
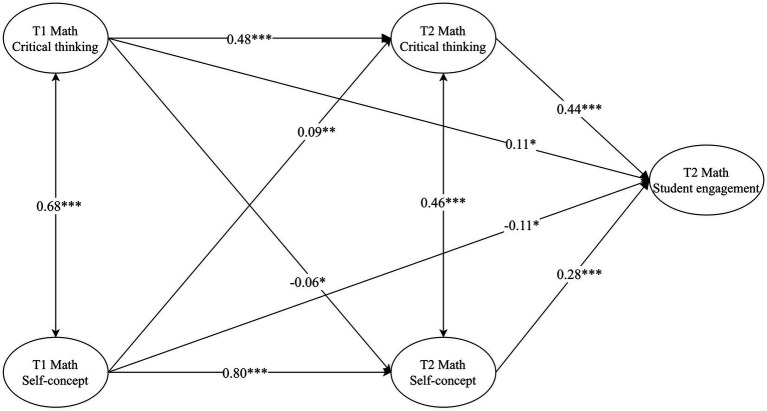
The model of the effects of Math critical thinking and academic self-concept on student engagement. ^*^p < 0.05, ^**^p < 0.01, ^***^p < 0.001.

The model fit the data well, *χ*^2^(284) = 1222.79, *p* < 0.001, RMSEA = 0.03, SRMR = 0.03, CFI = 0.98, TLI = 0.97 (see Figure 1). The autoregressive paths indicated that T1 academic self-concept positively predicted T2 academic self-concept (*β* = 0.80, *p* < 0.001). T1 critical thinking also positively predicted T2 critical thinking (*β* = 0.48, *p* < 0.001). The longitudinal paths showed that T1 critical thinking positively predicted T2 student engagement (*β* = 0.11, *p* = 0.001), but negatively predicted T2 academic self-concept (*β* = −0.06, *p* = 0.026). T1 academic self-concept negatively predicted T2 student engagement (*β* = −0.11, *p* = 0.019), but positively predicted T2 critical thinking (*β* = 0.09, *p* = 0.001).

#### 3.4.2 English model

**Figure 2:**
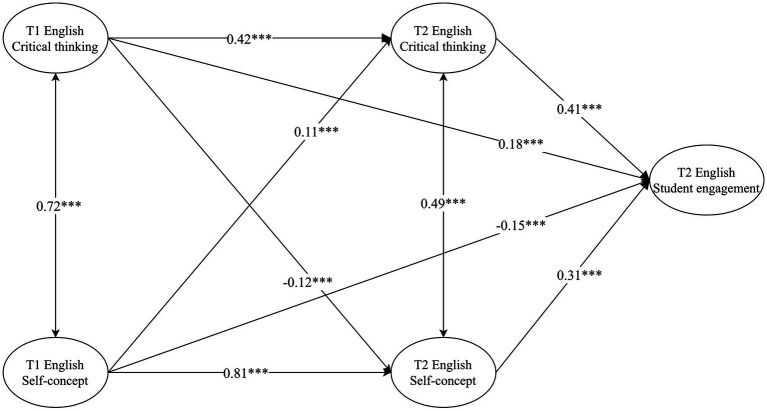
The model of the effects of English critical thinking and academic self-concept on student engagement. ^*^p < 0.05, ^**^p < 0.01, ^***^p < 0.001.

The model fit the data well, *χ*^2^(286) = 1293.06, *p* < 0.001, RMSEA = 0.03, SRMR = 0.03, CFI = 0.98, TLI = 0.97 (see Figure 2). The autoregressive paths indicated that T1 academic self-concept positively predicted T2 academic self-concept (*β* = 0.81, *p* < 0.001). T1 critical thinking also positively predicted T2 critical thinking (*β* = 0.42, *p* < 0.001). The longitudinal paths showed that T1 critical thinking positively predicted T2 student engagement (*β* = 0.18, *p* < 0.001), but negatively predicted T2 academic self-concept (*β* = −0.12, *p* < 0.001). T1 academic self-concept negatively predicted T2 student engagement (*β* = −0.15, *p* < 0.001), but positively predicted T2 critical thinking (*β* = 0.11, *p* < 0.001).

## 4 Discussion

This study examined cross-lagged relationships between critical thinking and academic self-concept and their longitudinal effects on student engagement among first-year university students. Overall, critical thinking and academic self-concept jointly shaped engagement, with similar patterns replicated across Math and English. Critical thinking positively predicted T2 engagement but negatively predicted academic self-concept; conversely, academic self-concept positively predicted subsequent critical thinking but negatively predicted engagement. These findings illuminate how cognitive abilities and self-evaluative processes interact during the transition to higher education.

### 4.1 Critical thinking and academic self-concept

This study revealed a reciprocal relationship between critical thinking and academic self-concept, supporting H3. Specifically, academic self-concept positively predicted subsequent critical thinking, consistent with prior research. As a motivational belief, academic self-concept shapes students’ cognitive resource allocation and strategy use, thereby promoting engagement in higher-order thinking processes such as analysis, reasoning, and evaluation (Wang and Wu, 2008; Guo et al., 2017). Students with higher academic self-concept also tend to report greater confidence and task value, which may further increase their willingness to engage in cognitively demanding activities (Dwyer et al., 2017).

However, critical thinking negatively predicted subsequent academic self-concept in both Math and English, a finding that diverges from previous studies reporting positive associations between the two constructs (Cormier et al., 2010; Chatzinikolaou and Tsirides, 2020). One possible explanation lies in the developmental characteristics of first-year university students. Although prior research has emphasized the analytical and regulatory functions of critical thinking (Facione, 2000), students at the beginning of university are still developing academic confidence and cognitive maturity (Hyytinen et al., 2018). As they encounter new academic standards, unfamiliar learning demands, and a more competitive peer context, stronger critical thinking may sharpen self-monitoring and increase awareness of the gap between current competence and university-level expectations. As a result, critical thinking may initially be accompanied by more critical self-evaluation rather than an immediate strengthening of academic self-concept.

A related explanation concerns the possible developmental mismatch between critical thinking and academic self-concept. Although both critical thinking and academic self-concept increased after university entry, their adjustment may not unfold synchronously during the first-year transition. Gains in critical thinking may prompt students to reassess their academic abilities before their self-evaluative beliefs become correspondingly stable and positive. This lack of synchrony may help explain why critical thinking showed a small negative longitudinal path to academic self-concept after accounting for stability paths and other variables.

### 4.2 Critical thinking and student engagement

Consistent with prior research, this study found that critical thinking positively predicted T2 student engagement, supporting H1 (Phan, 2009; Stupnisky et al., 2008). This result accords with social cognitive theory, which posits that higher-order cognitive abilities facilitate active involvement in learning. One possible explanation is that critical thinking enhances students’ capacity for independent reasoning, reflective judgment, and problem solving, thereby promoting deeper cognitive engagement (Manousou, 2025; Huang et al., 2025). Another explanation is motivational: students with stronger critical thinking are more likely to adopt mastery-oriented goals and value self-development, which may further increase their engagement in learning activities (Phan, 2009).

### 4.3 Academic self-concept and student engagement

A well-developed academic self-concept is typically linked to positive academic outcomes, including higher achievement, better learning strategies, more accurate self-evaluation, and stronger student engagement (King and Mcinerney, 2014; Marsh and Scalas, 2011). However, contrary to prior research, this study found that academic self-concept negatively predicted T2 student engagement, failing to support H2. Although the magnitude of this effect was small, its statistical significance warrants cautious interpretation in terms of practical relevance. A possible explanation lies in the person-environment fit model (Chatman, 1989). For first-year university students, the transition to university involves major changes in academic, psychological, and social demands (Hands et al., 2025). During this period, students may struggle to form stable self-evaluations, while the demands of adaptation consume substantial self-regulatory resources, thereby reducing engagement (Thau and Mitchell, 2010). Evidence from Chinese higher education contexts further suggests that students’ sense of belonging is closely associated with motivation, academic self-assurance, academic participation, and retention, underscoring the broader psychosocial adjustment involved in this transitional period (Rehman et al., 2023). Under such conditions, academic self-concept may show a short-term negative effect on subsequent engagement. This effect may diminish over time. As students adapt to the university environment, their academic self-concept becomes more stable and accurate (Gorges and Hollmann, 2019). In line with this interpretation, academic self-concept significantly improved over the first year in this study. As a result, its influence on engagement may gradually shift in a positive direction. Accordingly, although the short-term effect is negative, the overall longitudinal pathway from early academic self-concept to later engagement may still be positive through its effect on subsequent academic self-concept (Guo et al., 2022). Another possible explanation concerns self-concept recalibration during the transition to university. After entering university, students encounter new academic standards, unfamiliar learning demands, and changed peer comparison contexts. Under these conditions, academic self-concept may not immediately translate into stronger engagement, because students may still be adjusting their self-perceptions and developing the self-regulatory strategies needed to sustain behavioral and emotional involvement in learning. Thus, the negative path observed in this study may reflect a context-specific adjustment process among first-year university students rather than a generally negative role of academic self-concept.

### 4.4 Disciplinary stability in critical thinking, academic self-concept, and student engagement

While prior research highlights disciplinary influences (Umarji et al., 2018; Weich et al., 2024; Susperreguy et al., 2018), the present comparisons between Math and English suggest that the core dynamic associations among critical thinking, academic self-concept, and engagement are largely stable across both subjects. Although disciplinary contexts may shape the expression of these constructs, their underlying mechanisms appear broadly similar, driven by intra-individual processes and social comparison.

## 5 Conclusion and implications

This two-wave longitudinal study clarifies the dynamic interplay of critical thinking and academic self-concept and their effects on engagement among first-year university students. It provides empirical support for social cognitive theory and extends the literature on student engagement that prior critical thinking positively predicted T2 student engagement underscores the importance of cultivating critical thinking as a means of enhancing engagement.

These findings have several practical implications. First, instructional practices should more explicitly target the development of critical thinking. Classroom activities can incorporate discussion, reflection, multiple perspectives, and higher-order problem solving to promote critical thinking and, consequently, student engagement. Second, higher education institutions should foster supportive learning environments that strengthen students’ sense of external support and facilitate the development of a more stable academic self-concept. This may, in turn, promote more adaptive motivation, greater confidence, and stronger engagement in learning. Third, the relative stability of the relationships among critical thinking, academic self-concept, and student engagement across Math and English suggests that interventions targeting these factors may have value across disciplinary contexts. Taken together, these findings highlight the importance of promoting critical thinking and strengthening academic self-concept in order to enhance student engagement among first-year university students.

This study has four main limitations. First, only two measurement waves were included, which limits the ability to capture longer-term developmental changes in the relationships among critical thinking, academic self-concept, and student engagement. Future research should employ extended longitudinal designs, such as tracking students from entry to graduation, to provide stronger evidence for developmental processes. Second, the sample was drawn primarily from a comprehensive university in China, which may restrict the generalizability of the findings. Future studies should include more diverse regions and age groups to test the stability of these relationships across populations. Third, the study relied primarily on quantitative and self-report data, limiting insight into the mechanisms underlying the observed associations. Moreover, because student engagement was measured only at T2, the paths from T1 critical thinking and T1 academic self-concept to T2 engagement should be interpreted as predictions of later engagement levels rather than changes in engagement. Future studies should measure engagement across multiple waves. Future research should combine qualitative methods and experimental approaches to examine these processes more deeply in specific educational contexts. Finally, although the cross-lagged model provided useful evidence of longitudinal associations, it is limited in its ability to control for stable between-person differences. In addition, some SEM paths showed sign reversals relative to the bivariate correlations, which may reflect suppression effects or unique net effects within the multivariate model. Future research should employ additional measurement waves and consider random-intercept cross-lagged panel models (RI-CLPMs) to better distinguish within-person processes from between-person differences and to enhance the reliability and interpretability of longitudinal relations.

## Data Availability

The datasets presented in this study can be found in online repositories. The names of the repository/repositories and accession number(s) can be found below at: https://osf.io/4frhc/overview?view_only=5717dc2fb5914b8aaef477b27e90ed46.
